# Cell-Free DNA Variant Sequencing Using CTC-Depleted Blood for Comprehensive Liquid Biopsy Testing in Metastatic Breast Cancer

**DOI:** 10.3390/cancers11020238

**Published:** 2019-02-18

**Authors:** Corinna Keup, Markus Storbeck, Siegfried Hauch, Peter Hahn, Markus Sprenger-Haussels, Mitra Tewes, Pawel Mach, Oliver Hoffmann, Rainer Kimmig, Sabine Kasimir-Bauer

**Affiliations:** 1 Department of Gynecology and Obstetrics, University Hospital of Essen, 45122 Essen, Germany; pawel.mach@uk-essen.de (P.M.); Oliver.Hoffmann@uk-essen.de (O.H.); rainer.kimmig@uk-essen.de (R.K.); Sabine.Kasimir-bauer@uk-essen.de (S.K.-B.); 2 QIAGEN GmbH, 40724 Hilden, Germany; Markus.Storbeck@qiagen.com (M.S.); Siegfried.Hauch@qiagen.com (S.H.); Peter.Hahn@qiagen.com (P.H.); Markus.Sprenger-Haussels@qiagen.com (M.S.-H.); 3 Department of Medical Oncology, University Hospital of Essen, 45122 Essen, Germany; Mitra.Tewes@uk-essen.de

**Keywords:** metastatic breast cancer, liquid biopsy, cell-free DNA, next-generation sequencing, circulating tumor cells

## Abstract

Liquid biopsy analytes such as cell-free DNA (cfDNA) and circulating tumor cells (CTCs) exhibit great potential for personalized treatment. Since cfDNA and CTCs are considered to give additive information and blood specimens are limited, isolation of cfDNA and CTC in an “all from one tube” format is desired. We investigated whether cfDNA variant sequencing from CTC-depleted blood (CTC-depl. B; obtained after positive immunomagnetic isolation of CTCs (AdnaTest EMT-2/Stem Cell Select, QIAGEN)) impacts the results compared to cfDNA variant sequencing from matched whole blood (WB). Cell-free DNA was isolated using matched WB and CTC-depl. B from 17 hormone receptor positive/human epidermal growth factor receptor 2 negative (HR+/HER2−) metastatic breast cancer patients (QIAamp MinElute ccfDNA Kit, QIAGEN). Cell-free DNA libraries were constructed (customized QIAseq Targeted DNA Panel for Illumina, QIAGEN) with integrated unique molecular indices. Sequencing (on the NextSeq 550 platform, Illumina) and data analysis (Ingenuity Variant Analysis) were performed. RNA expression in CTCs was analyzed by multimarker quantitative PCR. Cell-free DNA concentration and size distribution in the matched plasma samples were not significantly different. Seventy percent of all variants were identical in matched WB and CTC-depl. B, but 115/125 variants were exclusively found in WB/CTC-depl. B. The number of detected variants per patient and the number of exclusively detected variants per patient in only one cfDNA source did not differ between the two matched cfDNA sources. Even the characteristics of the exclusively detected cfDNA variants in either WB or CTC-depl. B were comparable. Thus, cfDNA variants from matched WB and CTC-depl. B exhibited no relevant differences, and parallel isolation of cfDNA and CTCs from only 10 mL of blood in an “all from one tube” format was feasible. Matched cfDNA mutational and CTC transcriptional analyses might empower a comprehensive liquid biopsy analysis to enhance the identification of actionable targets for individual therapy strategies.

## 1. Introduction

Liquid biopsies harbor great potential for personalized treatment strategies and real-time monitoring approaches. In oncology, cell-free DNA (cfDNA) and, specifically, cell-free tumor DNA (ctDNA), defined by the presence of variants [1], as well as circulating tumor cells (CTCs), are powerful tools to describe tumor heterogeneity and clonal evolution [2]. 

High levels of ctDNA were significantly correlated with decreased overall survival (OS) in breast cancer (BC) [2] and, more specifically, *ESR1* cfDNA variants were associated with a shorter duration of endocrine treatment effectiveness in metastatic BC (MBC) [3]. Since patients with *ESR1* variants were described to benefit from fulvestrant rather than from exemestane, compared to patients without this alteration, *ESR1* variants also have the potential to tailor treatment regiments [4]. For disease monitoring, cfDNA concentration was shown to indicate impending relapse of primary BC earlier than any other imaging or blood-based strategy [5] and can predate treatment response changes [6,7]. 

The prognostic value of CTCs was first introduced for MBC patients more than a decade ago [8]. A decreased CTC count after treatment was significantly associated with increased progression-free survival (PFS) and OS [9]. Consequently, CTC count dynamics were proposed to be more suitable for monitoring than radiological imaging [10]. In addition to CTC counts, the expression profiles of CTCs were correlated with response evaluation criteria in solid tumors (RECIST) [11,12]. Positive results for therapy decisions in BC based on the CTC count, dynamics, or molecular characteristics, however, have until now not been convincingly examined in large randomized blinded clinical studies, but great efforts are underway to prove the predictive value of CTCs [2].

CfDNA and CTCs isolated from the same liquid biopsy specimen can enable comprehensive results if seen as providing complementary, rather than competitive information. CtDNA, released mostly passively, might present dying and probably therapy-sensitive cells [5]. On the other hand, CTCs are viable cells actively migrating into the circulation as potential seeds of metastasis and, therefore, possibly indicating minimal residual disease. It is not only possible to conduct mutational analysis of cfDNA and transcriptional analysis of CTCs using both analytes in parallel, but it was already described for multiple myeloma that sequencing of cfDNA and CTCs uncovered more mutations than analysis of either analyte alone [13]. Therefore, one might assume that parallel cfDNA and CTC analysis is complementary rather than competitive or even redundant [14].

Practically, however, many studies comparing cfDNA and CTCs used the same patient inclusion criteria, but different large cohorts for either cfDNA or CTC analysis [15,16]. There were a few publications analyzing both cfDNA and CTCs from the same patients, but the cohort sizes were quite small and the two analytes were obtained from blood samples taken at different time points [15,16]. Even if the study design considered the same time points for withdrawal, different preservative blood tubes, e.g., Streck and/or CellSave tubes, were mostly used [7,15,17,18,19]. In contrast, EDTA blood enabled parallel analysis of cfDNA and CTC from the same blood sample [20,21,22,23], but different blood aliquots were needed for both analyses, such that the required blood volume was around 20 mL [24]. Consequently, for appropriate comparability and consistency, the usage of the same blood sample with minimized volume drawn and stored/shipped under the same conditions for isolation of both analytes to reach an unbiased comprehensive liquid biopsy in an “all from one tube” format would be desirable.

We here (1) compared the quantity and characteristics of cfDNA variants isolated from whole blood (WB) and blood after positive immunomagnetic selection of CTCs (CTC-depleted (depl.) B), and (2) studied the heterogeneity of cfDNA variants and CTC overexpression signals in an hormone receptor positive/human epidermal growth factor receptor 2 negative (HR+/HER2−) MBC cohort.

## 2. Results

### 2.1. Patient Characteristics

The cohort was specified to only consist of patients with MBC with HR+/HER2− primary tumors. The majority of patients were more than 50 years old, and the median follow-up time was 68 months (range: 12 to 322 months). Eleven of the seventeen patients were deceased at the time of analysis. At the time of blood draw, the majority of patients had secondary metastases, received more than three treatment lines, and exhibited progressive disease according to response evaluation criteria in solid tumors. All patient characteristics are listed in Table S1 (Supplementary Materials).

### 2.2. Cell-Free DNA Concentration and Fragmentation

**Figure 1:**
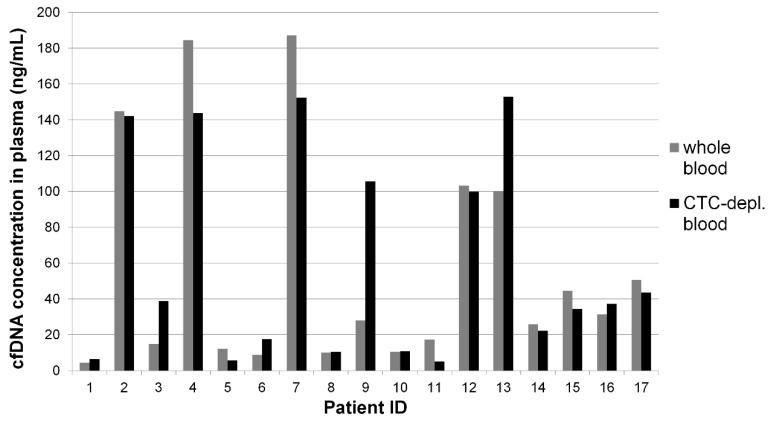
Inter-individual variability of cell-free DNA (cfDNA) concentration. Plasma from circulating tumor cell (CTC)-depleted (depl.)_blood (by AdnaTest EMT-2/StemCell Select) and matched plasma from whole blood were used in the same volume for cfDNA isolation with a QIAamp MinElute ccfDNA Kit, and cfDNA was quantified using an Agilent High Sensitivity Chip (fragments between 100–700 bp).

The used volume of the matched plasma samples was identical and ranged from 2.8 mL to 6.0 mL (mean 4.4 mL). Cell-free DNA concentrations in the plasma of WB ranged from 4 ng/mL to 187 ng/mL (mean 58 ng/mL), while cfDNA concentration in plasma of CTC-depl. B ranged from 5 ng/mL to 153 ng/mL (mean 61 ng/mL). Wilcoxon signed-rank test considering all 17 matched samples revealed no significant difference of cfDNA concentration in the different plasma samples. However, in some matched samples, the cfDNA concentration differed greatly and, importantly, a high inter-individual variability was observed (Figure 1).

**Figure 2:**
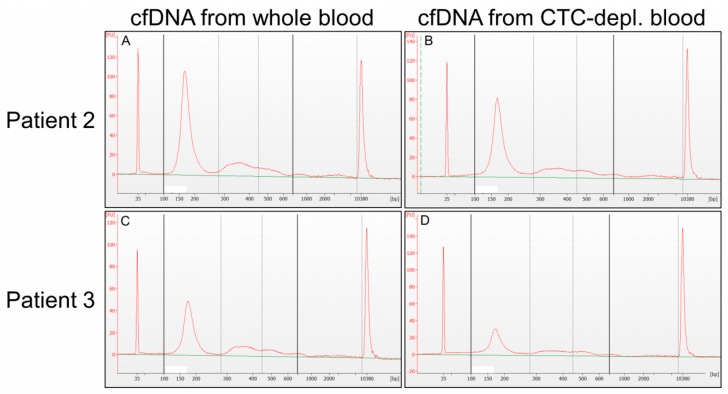
Size distribution of cfDNA. Matched cfDNA samples isolated from CTC-depl. blood (**B**,**D**; CTC isolation using AdnaTest EMT-2/StemCell Select) and from whole blood (**A**,**C**) of two exemplary patients (**A** + **B**; **C** + **D**) displayed a large mononucleosomal fraction and, in general, a similar size distribution without high-molecular-weight DNA (700–10,000 bp). Capillary electrophoresis was performed with an Agilent High Sensitivity Chip. (A) Cell-free DNA eluate from whole blood of patient 2, diluted 1:40; (**B**) cfDNA eluate from CTC-depl. blood of patient 2, diluted 1:40; (**C**) cfDNA eluate from whole blood of patient 3, diluted 1:5; (D) cfDNA eluate from CTC-depl. blood of patient 3, diluted 1:20.

Capillary electrophoresis using the Agilent High Sensitivity DNA Chip resolved the fragment length of the cfDNA. The majority of isolated cfDNA of all patients consisted of mononucleosomal cfDNA fragments (100–280 bp), but cfDNA fragments with a length of 280–450 bp/450–700 bp (di- or trinucleosomal DNA) were also detectable. Size distribution was comparable in cfDNA samples from WB and CTC-depl. B, as depicted for two exemplary patients (Figure 2). There was no difference in high-molecular-weight DNA (700–10,000 bp) in cfDNA eluates of the two plasma sources.

### 2.3. Cell-Free DNA Variants from Matched Whole Blood and Circulating Tumor Cell-Depleted Blood

Targeted deep sequencing was conducted for all cfDNA libraries >4 nM, resulting in removal of two matched cfDNA samples from one patient (Table S2, Supplementary Materials). Sequencing quality was guaranteed by exclusion of samples with <5 or >100 million read fragments, <400 unique molecular indice (UMI) coverage, and if <95% of the target region was covered with at least 5% of the mean UMI coverage, causing four samples and their matched samples to be rejected from analysis. The mean read count of all remaining samples from twelve patients was 12.41 million, while we found a mean of 3.5 reads per UMI and an average of 99.58% of the target region was covered with a least 5% of the mean UMI coverage (of 2760) (Table S2, Supplementary Materials).

**Figure 3:**
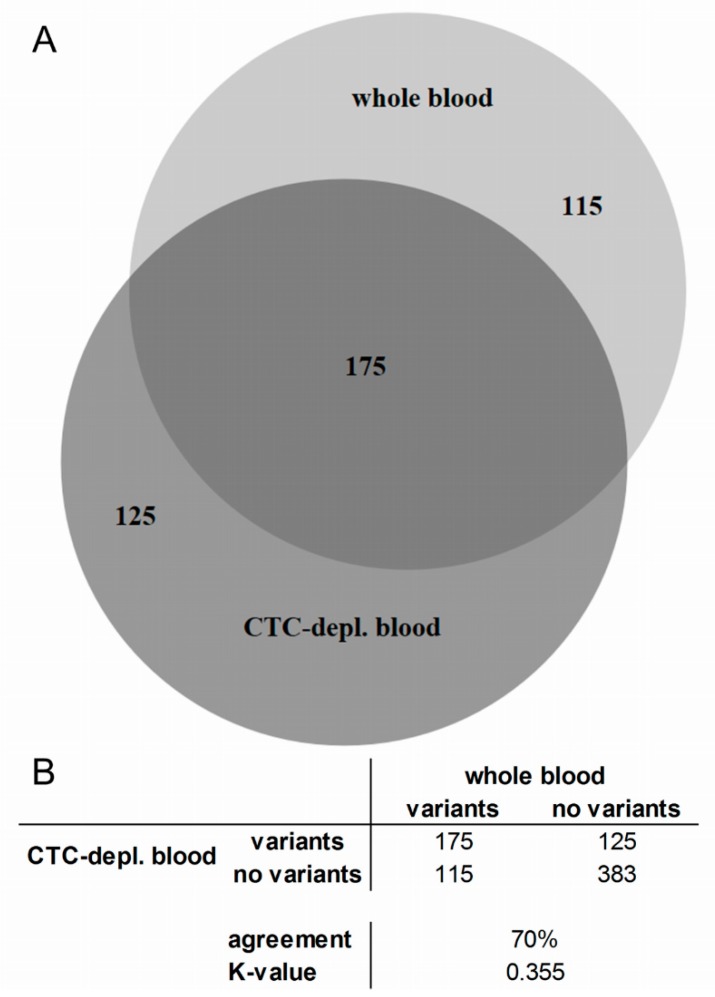
Venn diagram (**A**) and cross table (**B**) of matched cfDNA variants isolated from whole blood (light gray) and CTC-depleted blood (dark gray). Variants in all exonic regions of 17 genes were examined in 12 metastatic breast cancer (MBC) patients with hormone receptor positive/human epidermal growth factor receptor 2 negative (HR+/HER2−) primary tumor. Depletion of CTCs was conducted using AdnaTest EMT-2/StemCell Select. Variant calling by verification of unique molecular indices and filter of the Ingenuity Variant Analysis were described previously [25]. The proportional Venn diagram was computed using the tool BioVenn [26] and displays a great overlap of identical variants found in both cfDNA sources.

**Figure 4:**
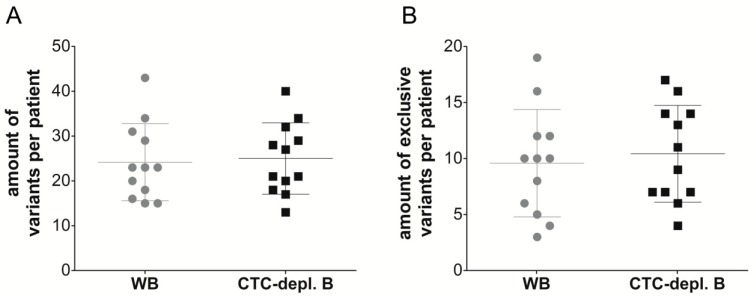
Boxplots describing the number of (**A**) detected cfDNA variants, and (**B**) the number of exclusively detected cfDNA variants per patient in whole blood (gray) and CTC-depleted blood (black, using AdnaTest EMT-2/StemCell Select). Means and standard deviations are also displayed, and the Wilcoxon signed-rank test indicated no significant difference between both cfDNA sources.

In total, 415 variants were detected by UMI verification and, after filtering with the Ingenuity software, in all 24 cfDNA samples from 12 MBC patients, 175 variants were identical in the cfDNA samples from both plasma sources, namely WB and CTC-depl. B (Figure 3A). Furthermore, 115 and 125 variants were exclusively found in WB and CTC-depl. B, respectively. Thus, the agreement was 70% with a *κ*-value of 0.355 (Figure 3B). Corresponding to the large overlap of identical variants found in both plasma sources depicted in the Venn diagram (Figure 3A), the number of detected variants per patient exhibited no significant difference between the two matched cfDNA sources (Figure 4A).

**Figure 5:**
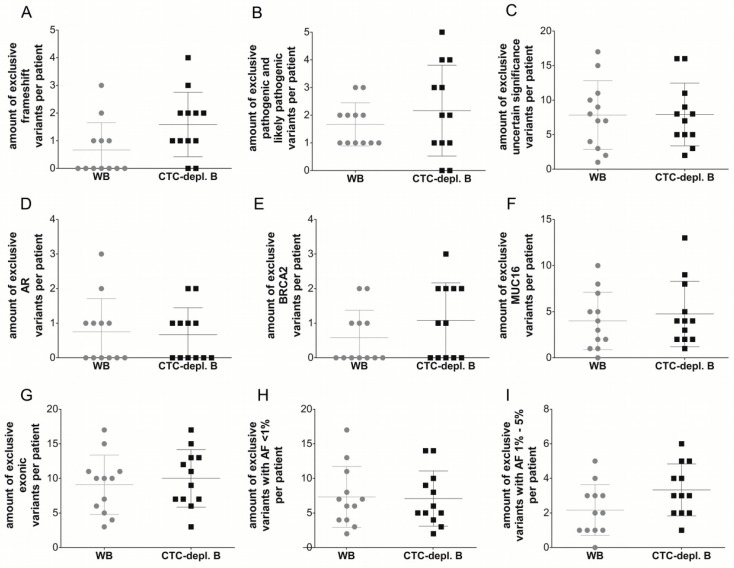
Boxplots describing the number of exclusively detected cfDNA variants per patient with specific characteristics in whole blood (gray) and CTC-depleted blood (black, using AdnaTest EMT-2/StemCell Select). The averages and standard deviations are indicated. Depicted characteristics are the major parameters of the categories: translation impact (frameshift (**A**)), classification (pathogenic and likely pathogenic (**B**), uncertain significance (**C**)), gene (*AR* (**D**), *BRCA2* (**E**), and *MUC16* (**F**)), gene region (exonic (**G**)), and allele frequency (<1% (**H**), 1–5% (**I**)). The Wilcoxon signed-rank test indicated no significant difference between the 12 matched cfDNA sources regarding any depicted characteristic.

Additionally, the number of exclusively detected variants per patient in only one cfDNA source was also not significantly different (Figure 4B). Despite the similar quantity of variants found in only one of the two cfDNA sources, the characteristics of these variants might be different. Therefore, the number of variants per patient with the most common characteristics in the categories: translation impact, classification, gene, gene region, and allele frequency (AF) was compared in matched samples (Figure 5). The average number of exclusive frameshift variants, *BRCA2* variants, or variants with an allele frequency between 1% and 5% was slightly higher in the cfDNA samples isolated from CTC-depl. blood, but the Wilcoxon signed-rank test indicated no significant difference between the number of exclusive variants with specific characteristics (frameshift, pathogenic and likely pathogenic, uncertain significance, *AR*, *BRCA2*, *MUC16*, exonic, AF <1%, AF 1–5%) isolated from WB compared to variants isolated from CTC-depl. B. Therefore, we concluded that cfDNA variants from matched WB and CTC-depl. B harbor no differences.

### 2.4. Clinically Relevant Characteristics of Cell-Free DNA Variants and Circulating Tumor Cell Expression

**Figure 6:**
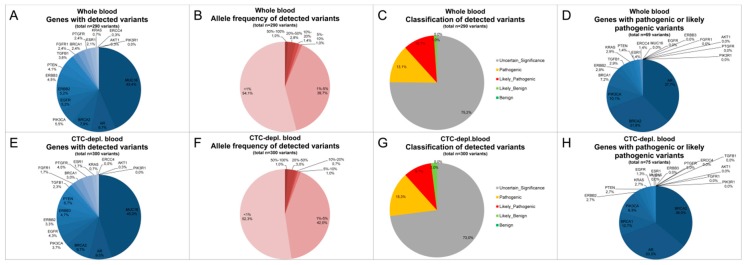
Distribution of called cfDNA variants isolated from whole blood (**A**–**D**) and CTC-depleted blood (**E**–**H**) of HR+/HER2− MBC patients (*n* = 12) according to their gene location (**A**,**D**,**E**,**H**), allele frequency (**B**,**F**), and classification (**C**,**G**). (**A**,**E**) Distribution of (exclusive (**D**,**H**)) variants according to their gene location. Most variants were found in the *MUC16* gene, while pathogenic or likely pathogenic variants were mostly located in the *AR* or *BRCA2* gene. (**B**,**F**) Allele frequency of all variants; 90% of all variants showed AFs <5%. (**C**,**G**) Classification of variants according to their known impact (benign, likely benign, uncertain significance, likely pathogenic and pathogenic) done by IVA. Nearly 25% of all variants are known to be likely pathogenic or pathogenic. No significant different distribution was detected for cfDNA variants from matched whole blood and CTC-depl. blood.

In the cohort of HR+/HER2− MBC patients, 50.0% of detected variants were located in the *MUC16* gene irrespective of the cfDNA source (Figure 6A,E). In total, 21.7%/22.5% of all variants isolated from WB/CTC-depl. B were located in the *AR* gene, and the third most commonly altered gene found was *BRCA2* (Figure 6A,E). Furthermore, 54.1%/52.3% of all variants had an AF of <1%, and 39.7%/42.0% of all variants isolated from WB/CTC-depl. B appeared with an AF of between 1% and 5% (Figure 6B,F). Moreover, 75.2/73.0% of all variants were of uncertain significance, but 23.8/25% of the variants isolated from WB/CTC-depl. B were already described to be either pathogenic or likely pathogenic (Figure 6C,G). Around 30% of these 69/75 pathogenic or likely pathogenic variants in WB/CTC-depl. B were located in the *AR* or *BRCA2* gene, followed by variants in *BRCA1* (Figure 6D,H). In summary, the distribution of cfDNA variants was similar in samples isolated from WB and CTC-depl. B with most variants having a low AF, being mostly of uncertain significance and being located in the *MUC16*, *AR*, or *BRCA2* gene.

To confirm that CTC analysis is feasible in addition to cfDNA analysis from the same blood sample, overexpression was examined by quantitative PCR (qPCR) in lysates of pooled CTCs. *mTOR* was the most commonly overexpressed transcript in CTCs of the studied cohort (Figure S1, Supplementary Materials). *AKT2* was also commonly overexpressed with a frequency of 88% in the cohort. *ERBB2, ERBB3, ERCC1, AURKA*, and *SRC* transcripts were overexpressed in the CTCs of more than 50% of all patients (Figure S1, Supplementary Materials).

## 3. Discussion

We here demonstrate that direct comparison of cfDNA variants isolated from matched WB and CTC-depl. B revealed no significant differences. The cfDNA concentration and size distribution, as well as the number of detected variants, were similar for both cfDNA sources. Even the characteristics of the exclusively detected cfDNA variants in either WB or CTC-depl. B showed no significant differences. 

### 3.1. Cell-Free DNA Sequencing from Circulating Tumor Cell-Depleted Blood Favored in the Future

The study was conducted to question the hypothesis that differences between WB and CTC-depl. B during cfDNA analysis occur, which were suspected to be caused by potential damage of CTCs and/or blood cells during CTC isolation, an influence of the immunomagnetic bead cocktail used for CTC enrichment, or an effect of additional 30-min incubation of WB at room temperature. However, since we found no differences in quantity and characteristics of the cfDNA variants isolated from WB versus CTC-depl. B, any systematic bias in sequencing cfDNA isolated from CTC-depl. B instead of WB can be excluded. Furthermore, we observed an unchanged sensitivity for cfDNA analysis using CTC-depl. B instead of WB. The blood supernatant remaining after positive immunomagnetic selection of CTCs using the AdnaTest EMT-2/StemCell Select procedure can, therefore, be used as source for an additional liquid biopsy analyte. Isolation of CTCs and subsequent cfDNA isolation from CTC-depl. B using the QIAamp MinElute cfDNA Kit, followed by UMI-confirmed sequence analysis in a combined workflow, provide the advantage of cfDNA mutation analysis in concert with a CTC molecular profiling from exactly the same blood sample. However, in contrast to the original AdnaTest protocol, 5 mL of WB turned out to be not enough to obtain a CTC-depl. B volume sufficient for the subsequent cfDNA analysis, because targeted PCR-based deep sequencing of cfDNA requires a high input amount of cfDNA. Consequently, the CTC isolation process was conducted in duplicate from 2 × 5 mL of WB to finally obtain minimally 4 mL of combined plasma from CTC-depl. B for cfDNA isolation with a yield >30 ng. Thus, we describe a workflow to isolate the two liquid biopsy analytes cfDNA and CTCs from a minimal blood volume of only 10 mL, resulting in reduction of the burden of blood-draw volume for the patient, while simultaneously empowering a comprehensive liquid biopsy analysis. 

### 3.2. Additive Value of Cell-Free DNA Mutational and Circulating Tumor Cell Transcriptional Analyses

A few studies comparing cfDNA and CTC data exist. However, in contrast to our present approach, for these studies, the blood volume needed for both analyses was additive and, thus, an additional burden for patients. Moreover, most of the isolation protocols required different preservative blood tubes for cfDNA and/or CTC storage. It was examined in BC patients that both total cfDNA level and CTC count were correlated with OS [17], that cfDNA integrity was correlated with CTC presence [20], that *SOX17* promotor methylation and *ESR1* methylation were highly concordant in ctDNA and CTCs [19,23], and that cfDNA and CTCs showed overlapping mutation profiles [17]. Using cfDNA variant analysis and CTC expression analysis of the same 10 mL of blood material, we here described the high prevalence of pathogenic or likely pathogenic *AR* variants (33.3%), and 38% of all 12 patients displayed an *AR* overexpression signal in CTCs. We conclude that AR might be of importance in HR+/HER2− MBC patients, both on a mutational and transcriptional level, as previously described by us in both cfDNA and CTCs in larger HR+/HER2− MBC cohorts [12,27]. Moreover, this gene is of relevance, because targeted treatment against AR is available; however, at the moment, it is only approved for other indications [28]. Furthermore, AR was described as a potential target in triple-negative BC patients [29,30].

Despite the similar results and consequences that can be drawn from cfDNA and CTC analysis as described above, cfDNA and CTCs also exhibit additive value. We here showed the high prevalence of *ERBB3* (70%), *ERBB2* (55%), and *PIK3CA* (27%) transcript overexpression in CTCs, whereas the prevalence of cfDNA variants isolated from matched CTC-depl. B and located in *ERBB3*, *ERBB2*, and *PIK3CA* genes was low (*ERBB3* 1.7%, *ERBB2* 2.2%, and *PIK3CA* 4.8%). This further exemplifies the additive value of CTC expression in combination with the cfDNA analysis. The protein expression of HER2 (encoded by *ERBB2*) is routinely used as a predictive marker for targeted therapy in BC [31]; therefore, the frequent overexpression of *ERBB2* transcripts in CTCs of patients with HER2- primary tumors, in line with previous results [12,32], might be relevant for treatment management in the future. Compared to cfDNA, CTCs provide a unique opportunity to study DNA, RNA, and proteins, even on the single-cell level, while also providing an indicator for active metastasis [33]. In particular, the expression analysis in CTCs was shown to be relevant for prognosis [34,35,36], prediction [37,38,39], and monitoring [11,12].

In contrast to CTC counts, ctDNA in MBC patients exhibited a greater correlation with tumor burden, ctDNA was more frequently found, ctDNA showed a greater dynamic range, and ctDNA described the earliest indication of response to chemotherapies [7]. Moreover, it was shown in BC patients that sequencing of cfDNA revealed more mutations than sequencing of CTCs [17], *ESR1* variant detection was more sensitive in cfDNA compared to CTCs [40], and cfDNA was correlated in a great extent with PFS than CTC counts [18]. Here, the existence of 1.3/10.7% pathogenic or likely pathogenic *EGFR*/*BRCA1* cfDNA variants accompanied by 0/25% overexpression frequency of matched transcripts in CTCs also highlights the value of cfDNA in addition to CTC profiling. *EGFR* variant detection in cfDNA was the first liquid biopsy test to be approved by the USA Food and Drug Administration, as a companion diagnostic in non-small-cell lung cancer patients [41].

In multiple myeloma, it was shown that sequencing of both cfDNA and CTCs uncovered more mutations than in either analyte on its own, as in some cases a subclone was only detected in one analyte [13]. Thus, not only does the combined analysis of cfDNA variants and CTC transcriptional profiles provide additive information, but the mutational analysis of CTCs and cfDNA—from now on feasible using the same 10 mL of blood—is also assumed to be complementary rather than competitive [14].

## 4. Materials and Methods

### 4.1. Patient Population Characteristics and Eligibility Criteria

The study was conducted at the Department of Gynecology and Obstetrics, in collaboration with the Department of Medical Oncology (for specimen recruitment), both at the University Hospital Essen, Germany, and in collaboration with QIAGEN GmbH, Hilden, Germany (for library preparation and sequencing analysis). In accordance with the Declaration of Helsinki, written informed consent was obtained from all participants at enrolment, and specimens were collected using protocols approved in 2012 by the institutional review board that consists of medical doctors, nurses, psychologists, welfare workers, and a priest (12-5265-BO). In total, cfDNA from 17 MBC patients was studied between October 2015 and June 2018. All participants were ≥18 years, had Eastern Cooperative Oncology Group (ECOG) scores for performance status of 0–2, no severe, uncontrolled co-morbidities or medical conditions, and no second malignancies. Prior neoadjuvant and adjuvant treatment, radiation, all kinds of surgical intervention, or any other treatment of BC was permitted. Patients had estrogen (ER) and/or progesterone (PR) receptor-positive primary tumors (summarized as HR+) and no ERBB2 overamplification (*n* = 16). Patients with ER-positive and/or PR-positive and HER2-negative metastases were also included if their ER, PR, and HER2 status in the primary tumor was unknown (*n* = 1). Patient characteristics are listed in Table S1 (Supplementary Materials).

### 4.2. Sampling of Blood, Isolation of Circulating Tumor Cells and Processing of Plasma

Initially, 2 × 9 mL of EDTA blood was collected in S-Monovettes (Sarstedt AG & Co, Nuembrecht, Germany), stored at 4 °C, and processed within 4 h of blood draw. Five milliliters of whole blood were used in duplicate to isolate CTCs by positive immunomagnetic selection targeting EpCAM, EGFR, and HER2 (AdnaTest EMT-2/StemCell Select, QIAGEN, Hilden, Germany), as described in detail elsewhere [42]. The remaining matched whole blood not used for CTC isolation was centrifuged at 3000× *g* for 8 min, and plasma was frozen at −80 °C. This plasma sample isolated straight from whole blood was abbreviated as WB. The CTC-depleted blood remaining after positive immunomagnetic selection, abbreviated as CTC-depl. B, was centrifuged at 3000× *g* for 8 min, and plasma was frozen at −80 °C.

### 4.3. Isolation of Cell-Free DNA

Matched plasma samples from WB and CTC-depl. B were thawed, centrifuged at 16,000× *g* for 10 min at 4 °C, and passed through a 0.8-µm pore size syringe filter (Sartorius, Goettingen, Germany). Cell-free DNA was isolated from 2.8–6.0 mL (mean 4.4 mL; maximized plasma volume available) plasma by affinity-based binding to magnetic beads according to the manufacturer’s instructions (QIAamp MinElute ccfDNA Kit, QIAGEN) and as described previously [25]. The same volume of plasma was used for isolation of matched cfDNA from WB and CTC-depl. B of the same patient. Cell-free DNA was eluted in 22 µL of ultraclean water and stored at −20 °C.

### 4.4. Cell-Free DNA Quantification

Diluted cfDNA (1:5 to 1:100) was applied to an Agilent Chip High Sensitivity DNA (Santa Clara, CA, USA). Concentrations of fragments with a length between 100 and 700 bp were summed using the 2100 expert software B02.08 (Agilent) to calculate the cfDNA yield.

### 4.5. Library Construction

The library was constructed with a customized QIAseq Targeted DNA Panel Kit (QIAGEN), as described in detail previously [25,27]. The input amount preferred for library preparation was in the range of 30–60 ng, but cfDNA samples with lower input were also included in the library preparation. The same cfDNA input amount of matched cfDNA samples from WB and CTC-depl. B was used for the library preparation, as listed in Table S2 (Supplementary Materials). Thus, the mean cfDNA input used across the cohort was 54.47 ng. We applied a 20 µL input volume in accordance with the protocol previously described [25]. Briefly, end-repair and A-addition was performed while the enzymatic fragmentation was inhibited. Subsequently, barcoded adapters including the UMI and sample-specific indices were ligated to the fragments. DNA was purified and free adapters were depleted by magnetic beads. The targeted enrichment was performed with a customized QIAGEN QIAseq Targeted DNA Panel primer designed to amplify all coding regions of the following genes: *AKT1*, *AR*, *BRCA1*, *BRCA2*, *EGFR*, *ERBB2*, *ERBB3*, *ERCC4*, *ESR1*, *KRAS*, *FGFR1*, *MUC16*, *PIK3CA*, *PIK3R1*, *PTEN*, *PTGFR*, and *TGFB1*. The universal PCR amplification was followed by a magnetic bead clean-up, and the final targeted enriched cfDNA library was eluted.

### 4.6. Sequencing

Libraries were quantified as published [25] by qPCR and the quality was checked using an Agilent Chip High Sensitivity DNA (Santa Clara). Libraries were diluted to 4 nM, and libraries with a lower yield (and matched libraries from the same patient; *n* = 1) were excluded. All pooled libraries were analyzed by paired-end sequencing on the Illumina NextSeq Sequencer with a NextSeq 550 System High-Output Kit, 2× 150-bp reads using a custom sequencing primer (QIAseq A Read1 Primer, QIAGEN).

### 4.7. Data Analysis/Bioinformatical Analysis

Data were initially analyzed using the QIAGEN GeneGlobe Data Analysis Center. Sufficient sequencing quality of all samples was guaranteed by exclusion of libraries with less than five million read fragments (*n* = 1), a UMI coverage lower than 400 (*n* = 1), and if less than 95% of the target region was covered with at least 5% of the mean UMI coverage (*n* = 1) [25,27]. Furthermore, libraries with >100 million read fragments were excluded to remove highly overrepresented libraries (*n* = 1). The matched libraries of the excluded ones were not analyzed to also guarantee comparison of only matched samples. The input amount, library yield, and sequencing quality parameter of each sample are summarized in Table S2 (Supplementary Materials). The QIAGEN Biomedical Genomics Workbench and the Ingenuity Variant Analysis plugin (IVA; QIAGEN) were used for further annotation, scoring, filtering (described previously [25]), and interpretation of variants detected in the UMI-based analysis. In detail, a consensus sequence was built from all fragments with the same UMI to exclude potential artefacts. 

The IVA used five different filters for exclusion of called variants. The confidence filter excluded all variants with a call quality below 20. Moreover, the confidence filter only kept variants that were not located in the top 5% of the most exonically variable 100-base windows in healthy public genomes. Variants with a prevalence of >0.5% in the normal population (reference databases: (1) Allel Frequency Community (gnomAD&CGI), (2) 1000 Genomes Project, (3) ExAC, and (4) NHLBI ESP exomes) were excluded, unless the variant was already known to be a pathogenic common variant, to identify rare variants potentially associated with the evaluated condition of the tested cohort (common variant filter). The genetic analysis filter only kept variants that were associated with gain of function or with the following inheritance patterns: homozygous, compound heterozygous, haplosufficient, hemizygous, het-ambiguous, or heterozygous. Only variants located no more than 20 bases from the intron were included, as well as those described to be pathogenic and/or likely pathogenic from the American College of Medical Genetics and Genomics, or variants that are loss-of-function-associated, which induce frameshift, in-frame indel, start/stop changes, missense, intronic two-bp splice-site loss, or any splice-alteration predicted by MaxEntScan (predicted deleterious filter). The cancer driver variant filter kept only variants that were found in (1) cancer-associated mouse knockout phenotypes, (2) cancer-associated cellular processes with any directionality, (3) cancer-associated pathways with any directionality, (4) cancer therapeutic targets, (5) published cancer literature variants and gene level findings, (6) known or predicted cancer subnetwork regulatory sites, (7) COSMIC at a frequency greater than or equal to 0.01%, and (8) TCGA at a frequency greater than or equal to 0.01%. 

Original raw sequencing data were uploaded as two fastq files plus MD5 checksum per sample, and are available at the European Nucleotide Archive with the study accession number PRJEB30449.

### 4.8. Messenger RNA Isolation and Quantitative PCR

Messenger RNA was isolated from the CTC lysates by oligo(dT)_25_-coated magnetic beads and was reverse-transcribed (Adna-Test EMT-2/StemCell Detect, QIAGEN; [42]). The AdnaTest TNBC Panel prototype (QIAGEN), consisting of multimarker real-time (RT)-qPCR assays, was applied to the complementary DNA isolated from the CTCs for expression profiling of *AKT2, ALK, AR, AURKA, BRCA1, EGFR, ERCC1, ERBB2, ERBB3, KIT, KRT5, MET, MTOR, NOTCH1, PARP1, PIK3CA, SRC*, and *GAPDH*, in relation to the leukocyte-specific transcript *CD45* and healthy donor controls. Experimental set-up und data evaluation were described in detail previously [12].

### 4.9. Statistical analysis

Statistical analysis was performed using SPSS, version 11.5 (IBM, Armonk, NY, USA). The Wilcoxon signed-rank test was used to assess whether the matched samples differed. Diagrams were computed with GraphPad PRISM (GraphPad Software Inc., San Diego, CA, USA) and Microsoft Excel (Microsoft Corporation, Redmond, WA, USA). Box plots display individual values for each patient, as well as the mean and standard deviation. The Venn diagram was produced with the online tool BioVenn [26]. The kappa-value was calculated using the GraphPad QuickCalcs website [43]. 

## 5. Conclusions

In summary, cfDNA mutational and CTC transcriptional analyses can supplement each other. A comprehensive liquid biopsy, therefore, increases the chance for identification of actionable targets to direct therapy strategies. Thus, the isolation of both CTCs and cfDNA, in an “all from one tube” format without bias due to the initial CTC isolation process, as demonstrated in this research study, might be helpful in paving a way for individual treatment decision-making.

## Supplementary Materials

The following are available online at http://www.mdpi.com/2072-6694/11/2/238/s1: Figure S1: Overexpression signal frequency in pooled CTCs all of blood samples (*n* = 12) subsequently called CTC-depleted blood and used for cfDNA isolation; Table S1: Patient characteristics; Table S2: Sequencing qualities.
