# Supplementary material for: Cell-Free DNA Variant Sequencing Using CTC-Depleted Blood for Comprehensive Liquid Biopsy Testing in Metastatic Breast Cancer
Source: Cancers (Basel). 2019 Feb 18;11(2):238. doi: 10.3390/cancers11020238 (PMC6406821; doi:10.3390/cancers11020238)

Figure S1: Overexpression signal frequency in pooled CTCs all of blood samples (*n* = 12) subsequently called CTC-depleted blood and used for cfDNA isolation. The relative frequencies of patients with HR+/HER2- MBC (*n* = 12), exhibiting CTC overexpression signals of the 17 assessed transcripts using the AdnaTest TNBC Panel prototype. 92% of all patients showed mTOR overexpression. *ERBB2*/*ERBB3* overexpression signals were present in CTCs of 55%/70% patients, respectively.

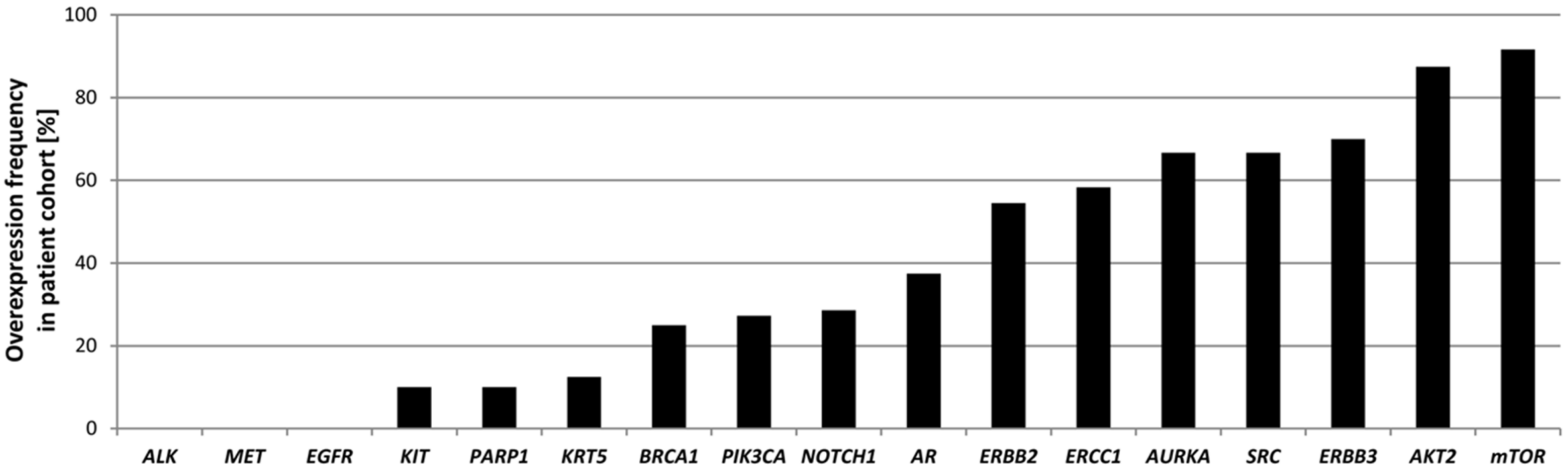

Supplement: Supplementary file 1 [file cancers-11-00238-s001.zip › Figure S1 Overexpression signal frequency in pooled CTCs.pdf]
